# A case study of the construction of inter-embedded multi-ethnic community of urban migrants in Nanyang

**DOI:** 10.1186/s41257-022-00076-7

**Published:** 2022-09-26

**Authors:** Qiang Sun, Dongxu Liu

**Affiliations:** 1 Institute of Ethnology and Anthropology, Chinese Academy of Social Sciences, Building 6, Yard 27, Zhongguancun South Street, Hai Dian District, Beijing, 100081 China; 2 Global Institute of Ethnology and Anthropology, School of Ethnology and Sociology, Minzu University of China, Nanrui Building, Yard 27, Zhongguancun South Street, Hai Dian District, Beijing, 100081 China

**Keywords:** Uygurs, Inter-embedded multi-ethnic community, Jade trade, Living around the market, Domestic population mobility, China

## Abstract

As the domestic interprovincial migrants, Uygur traders have been engaged in the Hetian jade trade from Xinjiang Uygur Autonomous Region to the city of Nanyang in Henan Province. Having lived in the private rented houses around the market in the early days, they moved into the government-built public rental housing in 2017, an independently managed community. They have maintained their past residential pattern of living around the market, and gradually develop the community into an inter-embedded multi-ethnic community together with other ethnic groups. Through the history of the formation of the Uygur community in Nanyang Stone Town, this paper presents the process of interaction between the ethnic minority migrants and the local community, examines the responses and influence of the market, society, government, and individuals in this process, aiming to understand how the minority community was formed, to deepen understanding of the “inter-embedded multi-ethnic community", and, in turn, to promote research and reflection on the development of the ethnic community during urbanization in China. This study suggests that such communities surrounding the market are socially extended based on inter-embedded livelihood. The open national market is the core of the social structure of inter-embedded ethnic groups, and market exchange is one of the main relations generated in the current interactions between the ethnic groups. The government’s efforts to build inter-embedded communities are conducive to transforming the relationships among ethnic groups from limited interaction to comprehensive and balanced development. Moreover, the improvement of common living environments and increase in exchanges are conducive to upgrading interactions between individuals to extensive and in-depth exchanges among different ethnic groups.

## Introduction

As China is in a transitional period from a traditional society to a modern society, its industrialization, marketization, and urbanization has led to large-scale social and geographic mobility within China. Since China’s reform and opening up in the late 1970s, it has been a distinctive feature of ethnic migrants that they form ethnic economy or migrant community in cities around a certain industrial chain. Examples of this include Ramen shops operated by the Hui from Qinghai, the sale of dried fruits, fruits and barbecued foods by the Xinjiang Uygurs, the sale of Buddha statues and ornaments by the Tibetans, and the sale of silver jewelry and Miao embroidery by the Miao. The focus of Xinjiang Uygur migrants on the jade trade is one of these examples. Through fixed and mobile sales, they have developed widely distributed jade sales networks in Xinjiang, Henan, Jiangsu, Zhejiang, and Guangdong, where they have gathered and formed residential areas, and interacted with the local communities, thus shaping the space for spontaneous interaction and exchange and intermingling of different ethnic groups (Liu, Sun 2018).

There are two basic frameworks with which to analyze such ethnic communities. One, which is mainly based on analysis of ethnic minority groups, believes that the closeness of the same ethnic minority group in terms of language, culture, and customs makes it easier for them to establish a group identity in a new city, with the bond of ethnic origin becoming an important social resource on which members can rely in the new environment. Uygurs in Beijing’s “Xinjiang Village", which has previously received attention from scholars, is a typical example of this (Yang, Wang 2008). While some scholars argue that the reliance on original relationships reflects the fact that ethnic minority groups in new cities cannot fully adapt to local life in a short period of time, others point out that this phenomenon may be a way in which ethnic minority groups can actively mobilize traditional resources to adapt to their new environments (Piao 2014). Either way, this analytical framework tends to view ethnic minority groups, the newcomers to the city, as relatively isolated, and differences in social life between them and the majority of the urban population are prominent (Zhou 2001). Another analytical framework favors the perspective of urban sociology, arguing that ethnic minority groups meet the special labor needs of some industries in the city, and that such special needs in turn prompt ethnic minority groups into cities to form new agglomerations in a new environment (Piao 2012, Liu 2013). Although these minority groups maintain a strong ethnic identity, they exhibit considerably different social and cultural characteristics from members of their ethnic groups in their hometowns. Once they have been integrated into a specific space, they form a community dominated by that ethnic group. However, this integration process is not as natural as is generally believed. Some studies have shown that in addition to the culture of the ethnic group, the economy (Bolt and Kempen 2010) and institutional environment (Musterd and De Winter, 2002) have a significant influence on integration. It is often the case that, with institutional acquiescence, ethnic migrants may overlap with other factors such as social strata, and then derive various forms of ethnic agglomerations (Liang 2011). The majority of mainstream urban research theories are positive towards ethnic communities that have developed diverse cultures during population mobility, considering them an important source of motivation for sustainable urban development. However, there is also a view that such ethnic communities may lead to spatial segregation of residences, which is of concern of those who argue that this spatial segregation may lead to urban fragmentation and management difficulties, and may increase psychological and emotional distance between members of society, constituting a potential cause of ethnic/group conflict (Hao 2012, Lai 2015). Therefore, they advocate that the government should plan and intervene in the spatial distribution of urban populations through various forms of community building, and this should become an important principle of contemporary urban governance.

Countries have taken different measures to address the issue in different countries based on their socio-historical backgrounds. Some countries and regions that advocate an “ethnic assimilation policy" regard the spatial scattering of ethnic minority groups as a means of achieving cultural assimilation, and better social integration is expected to be achieved by realizing scattered residence of ethnic minority groups with a series of strong government interventions such as allocation of public housing. Some countries that advocate “multiculturalism policies" consider social integration and cultural diversity conservation to be one process, attempting to achieve spatial integration of residences of different ethnic groups through socio-economic integration by improving the economic income levels of minority groups (Harrison, Phillips 2005). As some other countries identify spatial segregation of ethnic groups as class division, efforts have been made to improve the economic situation of members in the society, thus changing their position in the spatial distribution of ethnic groups, and ultimately weakening spatial segregation of residences based on characteristics of ethnic groups (Hao 2016).

As a unified multi-ethnic country with a long history, the People’s Republic of China faces this issue with its own situation. For many years, different ethnic groups of China generally choose to live among each other, while some live in concentrated communities of their own, and have a natural population mobility. As urbanization progresses, the prominent phenomenon of ethnic minority migrants gathering and living in cities has posed new urban management issues for governmental departments at all levels, and has also gained the attention of and prompted discussion by the academic community.

In response to these new phenomena, the Chinese government has put forward a new focus on ethnic work since 2010, to promote interaction, communication, and exchanges among ethnic groups, complying with the consistent advocacy of the Communist Party of China for ethnic unity. The Central Conference on Ethnic Affairs held in response to the new issues facing urban ethnic work in 2014 emphasized that focus should be placed on communities, as well as the development of inter-embedded multi-ethnic social structure and community environment^1^. The relevant guiding spirit was further emphasized in the 19th National People’s Congress in 2017 and the Central Conference on Ethnic Affairs of the Communist Party of China in 2021 (Xi 2018).

The phenomenon that large-scale population mobility leads to broad interactions between different ethnic groups since the reform and opening up has long received attention from Chinese academics. In the early stages, focus was placed on economy, employment of the migrants. However, an increasing number of studies have shifted their focus to ethnic groups with diverse cultures settling down in urban communities, which is an increasingly common occurrence.

The concept of “inter-embedded multi-ethnic communities" is an attempt to conceptualize this phenomenon. Some studies have spatialized the concept of inter-embedded multi-ethnic communities, emphasizing the hybrid nature of living spaces of different ethnic groups and arguing that a change in hard environment will inevitably promote interaction and integration (Zhang 2015). However, spatial “symbiosis" and “interactivity" are only one dimension of the concept of “embeddedness" Huang (2016), and it is unknown whether it necessarily affects other dimensions. The ideal state of “inter-embedded multi-ethnic communities" involves more dimensions and layers^2^. Therefore, more scholars believe that “inter-embedded multi-ethnic communities" should include multiple meanings (Wang, Yan 2015), and some scholars summarize them into three layers: housing pattern, social interaction, and spiritual culture (Zhang 2017). While this analysis is a valuable reference for understanding the meaning of inter-embedded multi-ethnic communities as a whole, it only reflects limited aspects of mutual interaction. Social and spiritual exchanges among members may manifest themselves in many other ways. It is more important to emphasize the interrelationship of the three layers from a dynamic, evolving and holistic perspective to understand the current multi-ethnic communities in urban areas, which have been formed through population mobility. Due to the strong mobility of the members of such communities, the residential pattern is not static. The initial residential pattern will change due to continuous interaction between the original community, migrant ethnic groups and local government.

The author began to pay attention to the Uygur migrant community in Nanyang City (in Henan Province, China) in 2014^3^, and has conducted fieldwork for six consecutive years since 2016. The fieldwork has mainly focused on Nanyang, where the Uygur community resides and conducts jade trade, and involved Hetian, Kashgar and Ili, the hometowns of Uygurs, as well as Suzhou, another major location in the jade sales network. Long-term studies have been conducted on the changes in Uygur production and life before and after migration, and the changes in the internal interaction of Uygurs and the interaction between them and the local community after their migration. Specifically, the author visited Nanyang in May and July 2016, Nanyang, Suzhou and Yiwu in May 2017, Urumqi, Hetian Region and Kashgar Region of Xinjiang in July 2017, Nanyang in April 2019, Urumqi and Ili Region in August 2019, and Nanyang again in June 2021, with over 10 months of field research in total. As we have kept in contact with the research subjects, we have used phone calls, WeChat calls, WeChat video interviews, live video streaming and emails to continue our research, conduct follow-ups and collect relevant information in recent years when field research could not be conducted due to COVID-19, in order to ensure the continuity of the study.

From the perspective of the formation and development of the Uygur jade trader community in Nanyang, this paper focuses not only on the influence of residential patterns on social exchanges and spiritual culture but also on the process of interaction between the original community, the migrant group and the local government. It also studies the influence of the interaction process on the three layers of interaction: residential pattern, social exchanges and spiritual culture. In the case shown in this paper, the influence has led to positive development in the business environment and community environment of the Uygur jade traders living around the market and developed a new outlook on the spiritual culture of the minority group and their social interaction with the locals.

## Research methods

This study mainly adopts ethnographic and anthropological methods for field research, through in-depth interviews, participating observation and small-scale seminars and other methods. We have conducted over 150 interviews with migrant Uygurs from Xinjiang engaged in the jade trade and relevant industries in Nanyang, more than 80 interviews with management staff of relevant governmental agencies in Xinjiang and areas to which Uygurs have migrated, more than 50 interviews with local residents, and over 40 interviews with non-Uygur origin jade traders; along with the interviews, we have compiled field materials of nearly 200,000 words. We have also observed the business operation and everyday life of Uygur migrants through field research, and compiled field materials of nearly 100,000 words. In addition, we collected relevant historical information, policy documents and other documentary materials. This paper is the stage achievement of the research.

The conclusion and discussion of this paper consist of five parts. Part 1 describes the background and scale of Uygur jade traders in Nanyang, a historic jade distribution center. Part 2 describes the formation of a specialized Hetian jade trading market for raw stones as Uygur jade traders have gathered in the Nanyang Stone Town. This market has transformed from an initial roadside market to a standardized indoor market, where an internal management mechanism of “middlemen" and “managers" emerged as the market started to be standardized, rather than loose or disordered as it was to begin with. Part 3 describes the shift of Uygur jade traders’ residence from rentals around the market to concentration in government- built public housing, a process that has enabled Uygur jade traders and their families to interact more with the locals; to some extent, the concentration is the beginning of a multi-ethnic community. Part 4 describes the development of this multi-ethnic community consisting of a vast majority of Uygur jade traders and their families, where the local government provides equal public services to residents and positive relationships and mutual recognition are created between Uygurs and locals through increased interaction and exchanges. Part 5 presents the difference between such structurally inter-embedded multi-ethnic communities centering on the market and the ethnic communities with spatial segregation of residence in the past; the inter-embedded multi-ethnic community is distinct as it involves comprehensive interaction in livelihoods, lifestyles and spiritual culture.

## Results and discussion

### The gathering of Uygur jade traders in Nanyang Stone Town^4^

Jade culture has a long history in China. In ancient China, jade culture served as a system of representation for the traditional political structure, and jade was endowed with great symbolic value. For this reason, the elite favored jade and thus substantially increased demand for it. While the jade used in the early stages was mainly produced on the Central Plains of China, jade materials from surrounding areas began to be discovered, transported and used after the dissemination of jade culture. Hetian jade soon gained the recognition of the governing class as it was transported to the Central Plains, which was the result of its embeddedness in ancient Chinese jade culture.

The city of Nanyang in Henan Province, located in the core area (the Central Plains) of ancient Chinese culture, has a thriving jade culture. It is famous for producing Dushan jade, one of the four most famous types of jade in China. Jade mining, processing and trading form the backbone of the local industrial chain. Zhenping County, under its jurisdiction, has emerged as a national hub for jade processing and trading that has taken advantage of geographical location and jade culture traditions since the beginning of the reform and opening up policy, and is known as China’s “hometown of jade carving". The Stone Town of Zhenping County is the core area of the jade industry, and has a total area of 148 square kilometers, which is mainly inhabited by Han Chinese, with a residential population of 72,000, nearly 80% of whom are engaged in jade or jade carving-related industries. Meanwhile, it is home to a large number of migrants from all over China, with a migrant population of 50,000. Since 2016, 10 large specialized jade markets have been formed, covering all links of the jade industry.

Xinjiang has a profound history of mining, processing and selling jade, which is represented by Hetian jade. Since the Yin/Shang dynasties, Hetian jade has flowed into the inland areas in large quantities in various forms, such as tributes, nongovernmental trade and official collection, forming a “jade road" connecting the Western Regions of China and the Central Plains. In the late 1970s, when the reform and opening up policy began in China, the jade and jade utensil markets became active. Some people from Zhenping County began to go to Xinjiang to purchase jade, and some of them established cooperative relations with local ethnic minorities, mainly Uygurs. Around the year 2000, a small number of Uygurs traveled to Zhenping County to sell Hetian Jade produced in Xinjiang. Since then, Xinjiang’s Hetian jade has risen in the national jade market, and attracted a large number of inland merchants to participate in Hetian jade trading. However, it difficult for inland merchants to travel to Xinjiang to purchase jade, and they faced procurement difficulties due to differences in language, culture and trading traditions. As an increasing number of Uygur traders realized the potential of the inland market, they started moving to the inland areas to trade Hetian jade. Once the Uygur traders became familiar with the inland market, they began to trade large quantities of Hetian jade to the Stone Town, and to Jiangsu, Shanghai, Zhejiang and Guangdong. They hereby formed a Hetian jade trading network led by Uygur traders crossing the eastern, central and western regions of China.

With the improvement of the business environment and living facilities in the Stone Town, some Uygur traders settled down here and have successively brought their families to live together from their hometown. They live in the Stone Town for business, and sometimes travel to coastal cities for trading. Thus, the Uygur population in the Stone Town has grown rapidly, and their businesses have extended from jade trade to catering, accommodation, transportation and other services. In 2007, the Hetian jade market saw a new wave of transactions since Hetian jade was chosen as the raw material for gold medals in the Olympic Games. In addition to being a high-grade decorative item, Hetian jade is considered an investment item, as many people are optimistic about its further appreciation, and this has inflated its price. The number of Uygur jade traders in the Stone Town has once again grown, with a peak of over 3,000 Uygur residents in the Stone Town around 2008 and more than ten thousand Uygurs operating on a short-term basis. However, since 2013, the number of Uygur jade traders in the area has fallen again as the overall jade market has cooled down. By the end of 2021, the Xinjiang resident population in Nanyang was around 1,000, with around 90% of these residents coming from various prefectures in southern Xinjiang, mainly Hetian, and a small number from Ili and Urumqi. 5

### Development and integration of Uygur jade market

The jade market in the Stone Town is divided into two main categories: indoor markets and roadside markets. Indoor markets include large and medium-sized jade malls and individual stores, which usually sell different grades of finished jadeware and jade ornaments. Road markets, on the other hand, are located on different sections of the road, depending on the types of jade and jade material being sold, with stalls selling raw stones, trimmings and relatively low-end finished jade pieces.

In the early days in the Stone Town, Uygur jade traders gathered in road markets around Zhaohe, Yufo Bridge and Yubo Road. Like local traders in the road markets, every day after the markerts opened, they would place the stones neatly on a four-sided cloth over the roadside, while waiting by their stalls for customers to come and ask for quotations, and making deals on the spot. The road markets were usually open for half a day, and when they closed, the traders would put the stones back into their sacks or customized iron cases and transport them back home in a trolley trailer or tricycle, waiting for the market to open the next day. As the Uygur traders would not return to Xinjiang for procurement until the stones were almost sold out, they would travel between the two locations at least two or three times, or up to seven or eight times per year. At the beginning, the jade market was rapidly rising, and market management was adapting to it. Market management was still immature, which was evident in the more mobile road markets. As newcomers to the market, Uygur traders inevitably encountered many difficulties in integrating into the local market, and an organizational mechanism was spontaneously developed to help them cope with and adapt to the local market. With strengthened management from the government on all jade markets in the Stone Town, the Hetian jade market dominated by Uygurs was re-planned and re-arranged, and significant changes have occurred in the residence and organization of the Uygur trader community.

#### The initial market (between 2000 and 2008)

In the early days, Uygur traders mainly sold Hetian jade. In addition to some jade collectors and investors, the majority of their customers were jade processors. These processors purchased raw jade, then made use of local jade processing techniques to create delicate finished jade pieces, which were then sold to other places. Uygur traders and local jade processors were situated in different links of the industry, relying on one another to complete the entire production and sales process.

Unlike ordinary commodities, jade has no standard market price. Although both sides of the transaction set prices based on their judgment of the jade quality, their judgement criteria and preferences may differ, so their expectations for prices may vary greatly. This makes bargaining one of the most important parts of the jade trading process. Bargaining requires face-to-face verbal communication between the two sides, with verbal communication being the most common channel. However, for most Uygur traders, communicating in Mandarin with locals who have Henan accents is often difficult, and many conflicts that occur in the trading process result from miscommunication. Therefore, many important transactions have to be carried out by Uygur middlemen who are familiar with local customs and have good language communication skills.

Although the quality of Hetian jade sold by Uygur traders varies, there are some high-quality jade pieces quoted with hundreds of thousands and even millions of RMB. In the period when the market was not sound in terms of technical conditions and lacked a modern market surveillance system, jade theft often took place. According to some jade traders interviewed, the market at that time was so crowded that it was difficult to obtain evidence and trace the theft cases. Usually, the investigation was not settled and Uygur traders often suffered losses as a result. Due to lessons learned after multiple thefts, they found that thieves were usually working in gangs. Once they were caught, their gang members would step in and interfere, which could easily turn into a group conflict. In 2006, local market regulators increased security patrols, installed surveillance cameras and further improved the regulation system, so that the theft of jade has been effectively curbed.

#### The organized market (since 2008)

Although controversial incidents arising from market operations over transactions have occurred from time to time, the market itself has developed in an organized and orderly manner, which is not only reflected in the further standardization of market management by relevant government departments, but also in the integration and organization within the Uygur Community. These Uygur traders, most of whom do not know each other, come from all over Xinjiang and gather in the Stone Town for business. Although they are of the same ethnic group, mutual discrimination and exclusion occurs due to geographical and urban/rural factors. As a result, the Uygur community faces difficulties in terms of getting along with the locals and handling internal relations.

As mentioned previously, most Uygur traders came to the Stone Town as individual vendors in the early days of the jade business. Although they shared the same market, their lives were not connected. This situation began to change with the emergence of some “leaders". Ike, from Ili, was one of them^6^. In approximately 2005, Ike came to the Stone Town to engage in the jade business. As he spoke Mandarin well, he often helped others to translate in the process of transactions. When disputes arose, he was asked to help resolve them. Gradually, he built up a reputation among the Uygur traders, and his gregarious personality helped him make many local friends. Historically, there has always been a tradition of “middlemen" mediating deals along the multi-ethnic ancient Silk Road, and when faced with the culturally diverse environment of the modern market, “middlemen” like Ike, who could reconcile the interests of all parties, were quickly recognized by Uygur businessmen. The government’s market authorities are in close contact with them, both to clarify market regulations to “middlemen" like Ike and to better maintain market order with their membership in the ethnic group. In 2010, the local market was further regulated when jade traders who had been working along the river and at the bridge were required to move to Yubo Road. Ike played an active role in assisting the relocation by paying for many jade shelves, which were placed along the roadside to facilitate the display of jade pieces, making stalls relatively fixed along the roadside market. According to the market management, each stall had to pay a daily fee of 3 yuan (RMB), which Ike helped collect, and this was used to hire someone to clean the market. Since then, payment for cleaning services indicated whether a businessman was officially accepted as a member of the market. The “middleman", represented by Ike, was given the role of “market manager". Although the management services at that time were limited in terms of efficiency, this was certainly the beginning of the integration of the Uygur market into a unified local management system.

For Ike, being a market “manager" not only meant having more social resources, but also being paid for helping others complete their transactions. As the market expanded, the number and frequency of Uygur jade traders travelling between Xinjiang and the Stone Town increased dramatically, and they began to travel to Suzhou and Yiwu to conduct business. As they had to carry large quantities of jade, it was important that they had easy access to transportation. As this was a business opportunity for Ike, he contracted several buses to provide dedicated shuttle services for traders who travelled weekly between different markets. With the convenience of road transportation, the Uygur jade trade has far exceeded the fixed jade market in the Stone Town, and evolved into a mobile jade trading network. Because the Stone Town has the largest jade trading base in China and its living cost is far lower than that in the southeast coast, people still choose to use the Stone Town as a transit station. Uygur traders like Ike saw this opportunity and began to explore services that could meet everyone’s daily needs.

As the main source of Hetian jade, the locals of Hetian in Xinjiang enjoyed unique advantages, and the number of Uygur traders of jade in the Stone Town was increasing. Hei Li (not his real name) was one of them. Having lived in the inland areas since the 1990s, he arrived in the Stone Town in 2009, demonstrating great ability in market management and communication. He called together a number of young Uygurs who had moved to the inland cities in previous years to help him, contracted the Longmao market next to the original Yubo Road market, and set up a new Uygur jade market exclusively for traders from Hetian. Meanwhile, he started a transportation business. Thus, the Yubo Road and Longmao markets, and the transport industry derived from them, began to be managed by Ike and Hei Li.

Although the government market authorities were clearly involved in the management of the Uygur market, the actual practice was to delegate the management to “middlemen’ from the Uygur trader community. As they were allowed to manage the market directly, they could dominate the market operation and the internal organization of the community. However, the change of the strength structure within the group would actually lead to the competition for the “power of management" in the market. Hei Li’s involvement was just the beginning, as jade traders Yimin and Mehmet, also from Hetian, showed their enthusiasm for market management. The two had also been recognized for their work for the locals. At the same time, Ike, the original market “manager", became involved in gambling and other bad habits, and his business and prestige fell. Seeing an opportunity, Yimin and Mehmet offered RMB 68,000 for Ike to transfer the “management" of the market. Ike took the money and left the Stone Town, never to be heard from again.

The Yubo Road area was part of the early market in the Stone Town, and the roads were narrow. With the development of the jade market on the road, especially after Uygur traders participated in the trading, the narrow market became even more congested. The crowd could only move slowly with the help of traffic police during the midday rush hour, which impacted the local traffic order and people’s normal lives. In April 2013, the local government re-arranged the jade market in the Stone Town, and moved the Uygur jade traders from the road market on Yubo Road and Longmao to the “World Jade Source Market" at the southern end of the International Jade City, solving the problem once and for all. The “World Jade Source Market" is divided into two halls, north and south, and each stall has been fitted with professional lighting equipment, making the market clean, spacious and bright, and greatly improving the business environment. Meanwhile, all stalls in the market are numbered in order to strengthen its standardized management, and each trader is assigned a fixed stall and operates with an authorized certificate. The hall is equipped with a comprehensive monitoring system, and professional security guards and Uygur joint defense guards for public security are employed to patrol the market, which has completely solved persistent problems such as jade theft, robbery and forced buying and selling. Adi and his wife operate two stalls in the North Hall, and in their 13 years of selling jade at Shifo Temple, they have witnessed huge changes in the area. “In the early days when we had stalls on the roadside, jade pieces were often covered by dust whenever a car passed by. There was no security guarantee or manager, but a lot of theft. In 2014, when we had just moved into this new market, there was a large surveillance system, and few people stole. In 2017, the government took back management of the market, and in 2018, cameras were installed at each stall. We seldom see any theft now, and even if there is a thief, they will get caught as the police arrive in 3 minutes”. In addition to the 478 Uygur jade traders in the new market, there are also dozens of Han Chinese merchants selling similar jade materials, forming an “inter-embedded" market in which people of various ethnic groups work together in jade trading. In the early days of the “World Jade Source Market", the “managers" of the market during the road market period secured the lease for the entire market from the developer, and then sublet each stall to jade traders, thus maintaining their control over the market. Later, however, the government of the Stone Town agreed with the developer that a corresponding agency would be set up to lease and directly manage the jade market, and employed Uygur jade traders such as Yimin and Mehmet to assist with market management. While these middlemen continue to perform their communication and coordination functions, the nature of their role in the market is completely different. The Uygur jade market has thus completed its transformation from a loose road market to a regulated indoor market, and has entered a new phase of development.

The market was the place where Uygur traders had the most contact with other groups, and market exchange relations were the first relationships formed between them and the basis for the expansion of their relationships with one another. Most of the disorder encountered by Uygur jade traders in their early days in the Stone Town was related to market disruption. The management of the jade market was loosely managed, and neither side of the trade was effectively protected, resulting in a spontaneous “middleman" management mechanism in the community. However, as the market’s hardware and facilities improved, the market management system was clarified and supervision was strengthened. Jade can be traded safely and smoothly, and conflicts have been significantly reduced. This shows that a well-regulated and orderly market is an important basis for good relations between different ethnic groups.

### Initial formation of the Uygur community

Large-scale population concentration along with the development of the jade market must catalyze various new social service demands, some of which can be met through the reciprocity of co-ethnics while more demands must be fulfilled by new community organizations and public service authorities in the city. On the one hand, the continual community of jade merchants and consumers has stimulated the burgeoning service industries of all types around the market. On the other hand, it has also prompted the government’s public service investment for this group and hereby promoted the formation of a multi-ethnic community dominated by Uygur families engaged in the jade business.

#### Ethnic aggregation during the period of the road market

When more Uygurs began to gather in Stone Town, their demands for different life services also gradually increased, drawing forth relevant service facilities. In the early stage, most Uygur merchants were engaged in a short-term mobile business model and mainly chose short stays in hotels around the county or market. However, their demand for accommodation rose when they brought their families to Stone Town. Many chose to rent local houses around Yubo Road and the jade market square where Uygurs ran businesses. While some lived in the same buildings as their landlords (but on different floors), more Uygurs rented entire residential buildings and sublet the the vacant rooms outside their own use to other Uygur businessmen. Due to dietary habits that differed from those of the locals, most Uygurs prepared dry food for themselves or went to the only Uygur restaurant in the county in the beginning. Soon, the restaurant opened a branch in Stone Town and offered accommodation. Several halal restaurants, Uygur naan bread bakeries, halal butcher shops and stores were subsequently opened around Yubo Road Market, turning the site into a center of Uygur life in the local area. This concentration on the market brought forth more social needs, the satisfaction of which in turn further facilitated assembly and integration of the community. Aini’s experience is a convincing example of this.

Born in Yili, Xinjiang in 1973, Aini has a childhood friend who is originally from Nanyang. This friend later followed his parents back to his hometown, but kept in touch with Aini. In 1994, Aini went to Nanyang at the invitation of his friend and happened to meet a family from Xinjiang who wanted to sell their restaurant. As Aini had worked as a helper in a relative’s restaurant for several years before, after a short investigation, Aini made a bold decision to acquire the restaurant with his friend and an employee of the restaurant, thence starting his business in Nanyang. At that time, his was the only Xinjiang restaurant in the county. Business flourished and passers-by from Xinjiang would occasionally visit his restaurant due to its reputation. As the jade market began to bloom, more and more Xinjiang natives came to Stone Town. At that time, the price of Hetian jade was still low. Some jade vendors would mortgage the jade at Aini’s restaurant at a relatively low price when they left and others would simply use the jade to cover the bill. Aini knew nothing about the quality or value of Hetian jade, but was so concerned about his “face" (means cultural understanding of respect, honor and social standing) that he often accepted the jade unselectively. Some time later, too many batches of Hetian jade piled up, affecting the circulation of the restaurant’s operating capital.

To turn the stock into cash flow for reinvestment, Aini attempted to sell the jade in Stone Town. Although sales were unsatisfactory due to the low quality of most of the jade, he saw huge market demand for Hetian jade as well as business opportunities here. Despite not being a good jade vendor, he was able to attract professionals from Xinjiang to sell Hetian jade here. The more people came, the better his business would be. Therefore, despite the opposition of his family, he went back to Urumqi and Hetian in 2003 to inspect the local jade market and spread the news that “it’s easy to make money in Stone Town” widely. When he returned to Nanyang, seven or eight jade vendors accompanied him to investigate the market. The first trip to Stone Town did not disappoint, and more and more Uygurs subsequently joined the jade vendors and conducted business in Stone Town. Aini’s news accelerated the connection between markets in Xinjiang and Stone Town, and many Xinjiang natives came to Stone Town due to his introduction. Later, he rented several houses on Yubo Road and opened restaurants and hotels in Stone Town, and many merchants would stay in his hotels and eat in his restaurants on business trips. Aini became a de facto key figure in guiding the ethnic group to take root in the local area at the early stage, as he provided everyone with a detailed profile of the local market and information about various social needs. His prestige gradually grew in the ethnic group. Aike, the interviewee mentioned previously, had benefited from Aini’s influence to some extent in becoming the “manager" of the market due to his role as Aini’s cousin.

#### The World Jade Source Market and the ethnic community

As one part of Stone Town’s overall jade industry and market planning, the World Jade Source Market is positioned as the exclusive market for the raw stone of Hetian jade (so-called seed material) with the aim of further standardizing and regulating the local jade industry. As most of the seed materials was operated by Uygur merchants, the majority in the World Jade Source Market are Uygurs who were previously scattered in all corners of the market due to its relative fragmentation. The building of the World Jade Source Market provided more possibilities for them to contact co-ethnic members, thus establishing more social ties and further strengthening internal integration.

The relocation of the jade business to the World Jade Source Market was inconvenient for Uygur merchants who lived around Yubo Road, 1.5 kilometers away from the Market. They had to drag iron boxes loaded with jade back and forth through the entire international jade mall every day, which was time-consuming and laborious. In response to the needs of all parties, following the standardization of the market, Zhenping County invested 41 million yuan to build the “World Jade Source" community in 2016, mainly provided public rental houses to non-local jade merchants including Uygurs. The community, which included a total of eight buildings with 408 sets of public rental houses, was equipped with community activity rooms, a supermarket, library, clinic, and other public facilities. In 2017, all Uygur families in the jade business who had been renting houses scatteredly happily moved to this new community, and their sense of happiness was greatly improved by the low rent and a livable community environment. Xiaoni, whose family was among the first batch to move into the new community, he said that “in the past, some could rent better dwellings while the others could only rent houses in poor condition. After the government built this community, everyone could live in an affordable place close to the market that had natural gas and a jade warehouse. It is so convenient for all of us.” It is notable that the originally dispersed residential pattern has been transformed into a centralized pattern during the standardization of the jade market, and that the economic subject relationship built on loose market relations had been transformed into a community member relationship.

The production and living needs of Uygur jade merchants were fully considered in the planning and construction of the World Jade Source community due to clear goals. First of all, given the business setting of the jade stall, the community was less than 200 meters from World Jade Source Market, only a five minute walk away, offering a convenient commute. Regarding vendors’ cutting and polishing needs, a processing area was specially placed at the back of the community. As it would be strenuous to carry large stones upstairs, a warehouse was placed downstairs in which everyone could store materials. Electric tricycles are crucial vehicles for all to deliver jade, so tricycle charging piles and parking lots were set up inside the community to facilitate residents. Secondly, given the significant characteristics of high mobility and various household types, the community housing was comprised of one-room flats, one-bedroom flat, and two-bedroom flats with accessible water, electricity, and gas. These layouts can meet the essential needs of everyone from a single businessman to a small family. Applications for moving in and out of the flat can be completed in the service center at the entrance of the community. Such arrangements can satisfy mobile vendors’ particular needs for short-term residence to the greatest possible extent. Thirdly, full consideration to security was given in its design, as the community is enclosed by walls and the gate is fitted with a monitoring device. Security guards are on duty 24 hours a day and visitors must register before entering the community. Such community regulation can guarantee safety of life and property for all residents.

In addition to hardware facilities, the local government also presided over the organization and construction of the community to ensure orderly administration. Since major residents of the community are Uygurs from Xinjiang, the community implements a “dual management system" under an effective exchange mechanism between governments of Xinjiang Uygur Autonomous Region and Henan Province. In general, the community work is led and coordinated by the Deputy Party Secretary of Stone Town and the heads of relevant local authorities concurrently serve as community leaders; on the other hand, cadres from Hetian, Xinjiang are dispatched to the workstation in Henan to assist with community management. Responsible for coordinating and handling various matters involving Uygur households in the community, the workstation staff have become an important force in community administration, relying on their language and cultural advantages. The workstation is generally managed by the head of station. This “dual management system" has effectively solved many flaws and misunderstandings in past migrant management and better guaranteed lives of migrant Uygur merchants here, thus posing great significance in promoting mutual exchanges. The community typically adopts a “Building Chief responsibility system", whereby each building chief is uniformly appointed by the workstation according to the actual situation. The Building Cief manages affairs of the entire building via activists on each floor and reports to the workstation.

Most residents used to live in rental houses separately and lacked communication in everyday life. They were not familiar with each other despite living in the same community. In order to promote mutual exchanges and enrich community life, the workstation made use of holidays and Uygur customs and organized a series of activities such as publicity of laws and regulations, singing competitions, and the Eid al-Adha. The second floor of the south hall of the market was arranged as the temporary venue of worship where Imam in charge of religious work was assigned by the Hetian government. It became more convenient for ethnic minorities to participate in religious activities in both the community and the market.

Several Xinjiang restaurants and supermarkets appeared in the surrounding area after the World Jade Source Market was put into operation, and the square in the middle of the two markets was also planned as the site for temporary stalls to open shops selling featured snack food. Shops along the street in the community were rented out to Uygur residents to sell fruits, vegetables and other goods. Some of these shops were relocated from Yubo Road while others flowed into the area later. New service facilities developed around the jade market and a multi-ethnic community gradually took shape around the market.

### Construction and development of the Uygur community

Although the building of the World Jade Source community has essentially solved the long-standing problem of inconvenient accommodation for all ethnic groups engaged in the jade industry, including the Uygurs, living is only one aspect of the community function. To enable ethnic minority groups to live in peace and work happily in a new environment, based on equitable public services to both Uygur jade merchants and their families and indigenous residents, the local government has actively promoted the exchanges and interactions among different ethnic groups by improving an equal public service system and carrying out ethnic unity activities. The main aspects concerning the construction and development of an inter-embedded multi-ethnic community include:

1. Equalizing public services catalyzed by the government

Preliminary interactions between Uygur jade merchants and the natives were quite limited as their ties were mainly built on market relations. When jade vendors shifted their business mode from solo mobile trade to family-based migrant operations, their demand for public services increased. Finding solutions to solve difficulties from their co-ethnics constrained their social scope and was not conducive to exchanges with other ethnic groups. To tackle this stalemate, the local government began with tackling the needs of Uygur families engaged in the jade industry and endeavored to provide available public services equal to those of indigenous residents to bolster the social integration of the migrant groups.

Access to equitable education for the school-age groups of the migrant population is not only a requirement by the law for citizens’ basic right to education, but also a crucial condition for propelling social relation restructuring and integration into the local society for the migrant population. Statistics from 2013 show that approximately 30% of the Uygur migrant population stationed in Stone Town were juveniles, of which dropout students took up a high proportion. The main reasons for the high dropout rate included language barrier as some migrant children could not speak the language, children being over the age of school enrollment, inability to provide household registration and school registration certificates, parents’ weak educational awareness and lack of educational competency, unstable residence, and other factors. To solve these problems, the local government formulated comprehensive measures to advance the school enrollment of migrant students. Clarifying working principles of “nearby schooling" and “centralized management", the government all Uygur school-age children to be “directly enrolled in the nearest school". Meanwhile, staff also visited and mobilized parents to support their children’s schooling; bilingual teachers were introduced and language training courses were held; “Nine Ones”^7^ service work was established; students were organized in mixed-ethnicity classes; thematic courses such as “Big Family of the Chinese Nation" and “General Knowledge of the Nation" conducive to national unity were run; a variety of training activities such as “one-for-one" tutoring and “many-for-one" tutoring were held to help Uygur students who had learning difficulties. With the goal of improving educational services, families of different ethnic groups were mobilized to promote educational integration in the local community and facilitate better integration of Uygur families into the local community (Sun 2018).

Everyday life services should be perfected to relieve people’s worries. Healthcare, transportation, and religious beliefs are important guarantees for Uygur families to integrate into local life. To make medical care more convenient for the Uygur people, hospitals at the county and town levels set up bilingual signs in Uyghur and Chinese. The township-level healthcare center has established a “green channel" for Uygurs and implemented a “one-to-one" barrier-free medical treatment model for Uygurs. The community health station was set up by the Community Party Service Center of the World Jade Source community in 2016 to provide services in the neighborhood. To make business travel more convenient for Uygurs, the county government invested 220 million yuan to build the only township-level bus station in Henan Province. The station is equipped with bilingual signs in Uyghur and Chinese and has become a hub linking Hetian, Zhenping, Suzhou and other places on the “Road of Jade". To protect the legitimate religious activities of the Uygurs in accordance with the law, a temporary religious site covering 500 square meters was set up on the second floor of the south hall of the World Jade Source Market in Stone Town in 2014, where patriotic Imams from the Hetian area of Xinjiang were designated for preaching and scripture interpretation to meet the standard religious needs of ethnic minorities.

Juridical support is in place to keep transactions safe and resolve disputes. To create a safe social environment for people of all ethnic groups, Stone Town has organized a PAP squadron and one public security bureau and deployed security personnel, physical security, and technological security facilities with the aim of building a comprehensive public security prevention and control system. People’s sense of security has been remarkably enhanced via science and technology in the whole market. Community workstation staff popularize legal knowledge at irregular intervals and Uygur businesspeople are encouraged to settle conflicts in everyday life and transactions through legal tools; a special team of over 40 fluent Mandarin speakers who are respected and reliable members of Xinjiang ethnic minorities has been established; a joint defense team for public security composed of 10 Han individuals and 10 Uygurs has been set up. These two teams actively deal with different contradictions and disputes between the Uygurs and the Han. Promptly eliminating hidden dangers, the success rate of mediation has reached 95%. Many conflicts and disputes are terminated at an early stage to avoid incidents that could jeopardize ethnic relations.

Emphasis is placed on public spiritual and cultural life. With “National Unity Month" as a vehicle, the community organizes a community talk every month to understand residents’ ideas, needs and difficulties. Meanwhile, public welfare lectures for which legal professionals are invited to explain various ethnic policies and spread legal knowledge are given. To enable Uygurs to better understand the development and changes in their hometown, the second set of Xinjiang Satellite TV programs (Uygur channel) was introduced in Zhenping County due to the efforts and coordination of multiple parties. The community also holds regular flag-raising ceremonies and launches a selection of “model families and model residents"; public art performances participated in by all ethnic groups are held. In accordance with the needs of ethnic minorities, national common language and diverse vocational training such as making pastry, sewing, and online live streaming are provided free of charge to advance Uygurs’ capacity for self-development while enriching their spiritual and cultural lives.

2. Inter-ethnic friendship cultivated in community life

Exchanges and interactions among different ethnic groups are embodied in a concrete inter-personal relationship, in which the common business and living circles serve as foundations for establishing close relationships between Uygurs and native residents. Although economic relation is the main form of relationship between the Uygurs and their neighbors, this initial interaction naturally evolves into more complex interactions as participants are members of the society. Going beyond economic ties, diverse relationships have developed through the market, some of which have negative aspects such as labeling and stigmatization; other experiences are positive, such as friendships gradually cultivated from initial business partnerships. Estrangement originally caused by incomprehension gradually disappears after more interactions. The government’s active intervention and guidance based on spontaneous interaction at the individual level could further accelerate mutual understanding, acceptance, and tolerance among all ethnic groups. More ways and wisdom of living together were explored while admitting the objective differences. This should be the meaning of realizing ethnic integration.

Uygur jade merchants encountered many difficulties in business and life in the early days. Some people described their feelings at that time: “I have no alternative but to sell stones here, but I don’t like living here. The locals are hard to get along with.” Correspondingly, some locals thought these Xinjiang people were generally “rude" and “difficult to communicate with". However, the survey indicates that although this situation did exist in early contact, the facts were exaggerated. In fact, Uygur merchants and the locals were not mutually exclusive from the beginning. On the contrary, many of them developed good relationships and affection in long-term contact. The oft-cited tensions are mainly derived from individual competition and conflicts in the period of non-standard market, which are somehow inappropriately labeled as ethnic-relations problems. With constant conceptual improvement in the local market management and perpetual advancement of technical methods, the jade market has become standardized and mature. Conflicts between groups in the market have become a thing of the past and various economic disputes can be effectively resolved through mediation or legal means under primary-level governance. Although memories of previous conflicts still influence different groups’ impressions of each other and communication to some extent, there are more cases of mutual acceptance and identification in everyday interactions.

The story of the jade merchant Abdu and Wang Peng vividly illustrates the emergence and continuation of cross-ethnic friendship through everyday interactions. The the locals in Stone Town are engaged in electric tricycle transportation, which is a very important transportation mode for Uygur jade merchants, because they often rely on these tricycles to transport tens or even thousands of kilograms of jade. In 2008, Abdu came to Stone Town alone, dragging hundreds of kilograms of jade. After getting off the shuttle bus, he happened to meet Wang Peng who was waiting for business. Abdu accepted his ride and their first contact on the small tricycles left Abdu with a good impression of Wang Peng. From then on, Abdu would hire Wang Peng when he needed to deliver the stones. He always respectfully called Wang “elder brother". An honest and trustworthy man, Wang would come to pick him up as long as he promised Abdu no matter it was windy, rainy, hot or cold, and he never charged him any extra. Later, they became stable business partners. In the beginning, business was difficult. Once, Abdu needed turnover capital so desperately that he seeked help from his elder brother to borrow money. Though Wang was not rich, he did not hesitate to lend thousands of yuan to help Abdu through this hard time. When Abdu was hospitalized due to an acute disease, Wang took care of him until he recovered. As a stranger on this land, Abdu was deeply moved by Wang’s trust and friendship witnessed by trials and tribulations. Later, Abdu’s business began to take off and he opened a jade shop. In 2016, Wang suffered a stroke and was suddenly in a plight. His child was still in elementary school and his wife left him. Abdu donated 45,000 yuan to his elder brother for treatment and often visited Wang in the hospital. After leaving the hospital, Wang could no longer work in freight transport due to his physical condition, so his source of livelihood was cut off. Abdu then hired him to help look after the shop and paid him 2,000 yuan per month. Abdu’s support reignited Wang’s hope. From mutual recognition and support in business to mutual assistance in life, two strangers from different regions and nationalities interpreted the exchange, communication, as well as integration in ordinary people’s everyday lives with their genuine friendship.

In addition to the close relationships established through the jade business, landlord-tenant relationships are a common form of relations between the Uygurs and the natives. Han Bin and the jade vendor Nyaz gradually evolved from landlord and tenant to close friends. Han Bin is representative of many local landlords. Running farmland and engaged in the jade business, he is also a landlord with four apartments in the town, one that he lives in himself and the others that he rents to Uygur merchants all year round. The Nyaz family had been living in his house for seven years before moving into the new community. The jade business was a risky one with many frauds. When confronted with a scam, vendors would be on the verge of bankruptcy. Nyaz was once a victim of a deception and could’t afford the rent for several months as a result. Instead of embarrassing Nyaz, Han told him that everyone encountered difficulties and that he could make up for it when things were better. Nyaz was very grateful for his trust and paid off the rent he owed as soon as possible. Since then, whenever there was a traditional Uygur festival, Nyaz would invite Han to his home and Han would gladly accept. Nyaz would also visit Han to drink and chat on holidays. In addition to selling jade, Nyaz raised pigeons and chickens to support the family when the jade business was not going well. Sometimes he would give Han livestock, and he maintained a good relationship with the neighbors. Han thought that “some locals are prejudiced against the Uygurs, but there are very few bad guys. In fact, the crux of the problem is lack of communication. I feel they are very genuine and not narrow-minded. If you respect them, they will in turn show more respect for you.”

The story of Xiao Ha, a young jade merchant, is a convincing one that reflects emotional integration among ethnic groups. Xiao Ha came to Stone Town for the first time in 2007. At this time, he was an 18-year-old teenager who yearned to change his destiny and family. It was a most unforgettable experience in his life, and he recalled: “My family was very poor when I was a child. I came here because relatives told me that it was easy to earn money here. It was difficult to find a place to live as I didn’t even have a cent. I met an old couple and asked them to rent to me a house at 5,000 yuan a year, telling them I had no money now but would give it back in the future. I didn’t expect them to say yes. Unexpectedly, they told me that ‘you can pay the rent if you have money, and there is no need to pay if you don’t. As a stranger, it's not easy to earn money here.’ I was so touched that I could never forget it for the rest of my life. Business has been going well in the past few years and I have earned some money here. This place is like my hometown and I want to devote myself to making it better.” A bond of deep friendship was forged between Xiao Ha and his landlord’s family. Xiao Ha also shows his love and warmth to this land by sponsoring local poor students.

The examples above profoundly demonstrate the earnest friendship between Uygur jade merchants and indigenous residents formed in a shared life owing to the development of jade business. While such emotions are usually hidden by the busy rhythm of work, they echo strongly during special events. During the national poverty alleviation campaign, some Uygur jade merchants learned that there were still poor farmers living in some mountainous areas of Nanyang. They spontaneously decided to donate money and necessities to these households to solve some difficulties of the poor. Later, the campaign was further promoted by the Xinjiang work team and attracted more competent Uygur jade merchants. Over 150 Uygur merchants generously donated more than 450,000 yuan to fund local Han people in need. They said that they had lived here for a long time and were quite willing to contribute to the local area in this way. The shift from individual mutual assistance to spontaneous group donation reflects the sublimation of emotional integration.

Nanyang, the city in which Stone Town is situated, sits in the Central Plains, the heart of Chinese culture from prehistoric period to Shang dynasty and the cradle of Hua-xia civilization, or Chinese civilization. The surrounding ethnic groups had continued to interact with residents of the Central Plains for a long time throughout history, with some of them organizing regimes after settling in the Central Plains. The history of exchanges and integration has generated a distinctive culture of integration, inclusiveness, and diversity in the Central Plains. Having passed through dynasties, this tradition continues to develop and shape cultural advantages attracting multi-ethnic groups in modern times. Historically, interactions between the Central Plains and different places of the Western Regions of China are explicitly reflected in the development and prosperity of the Jade Road and the Silk Road, and the interactions among ethnic groups in the Western Regions and the Central Plains set an important foundation for the rich connotations of Chinese culture. Therefore, the Uygurs, despite their seemingly significant cultural and conventional differences from the native residents, are able to live and work in Stone Town within a short period of time and develop deep friendships with other residents of ethnic groups in everyday life and business. On the one hand, this can be attributed to the inclusive nature of the traditional Central Plains culture; on the other hand, it is a natural extension of longstanding inter-ethnic exchanges among the Central Plains and different ethnic groups of the Western Regions throughout history.

### Living around the market: an inter-embedded multi-ethnic community

From the above analysis, it is clear that the aggregation and development of the Uygur ethnic group in Stone Town is directly related to the jade market. Nanyang is renowned for producing Dushan jade and possesses a long tradition of mining, processing, and selling jade. Since the 1980s, in addition to the collective operation mode of jade processing enterprises, self-employed households based on family workshops developed rapidly. Nanyang has gradually become one of the largest comprehensive jade processing bases and trading centers in China as the reputations of its local jade processing and trading market have grown nationwide. Due to the varied characteristics of jade, processing technology, sales objects, and trading channels differ for various types of jade. Therefore, a relatively independent secondary market network has been formulated based on varying jade types inside Stone Town’s large jade market. These secondary markets have unique characteristics and complement one another, satisfying all kinds of customer needs and jointly contributing to market prosperity. Hetian jade, one of the four famous types of jade, is an indispensable part of the market. The local industrial chain of Hetian jade came into being after the 1990s. Uygur merchants were transport and sales agents for raw Hetian jade stones. Their strengths in region, language and culture enabled them to act as irreplaceable market participants. On the other hand, their advantages could not be exerted without this special market structure, let alone their aggregation and development in the local area.

The concentration of Uygur merchants changed along with the gradual development of the jade market in Stone Town. All market subjects were flexible in the road market period, there was no standardized market regulation and most Uygur merchants opted to temporarily rent around the market for convenient business-running purposes. A loose dwelling area around the jade market was hence established. With gradual standardization of the jade market, most stalls on the road market were moved indoors, the large jade market was planned and arranged to high standards and the market for Hetian jade seed material was uniformly integrated into the new World Jade Source Market. In order to facilitate the business operation and life of Uygur merchants, the government planned and built a public rental housing community for Uygur merchants near the new market. Tenants of public rental housing are only entitled to temporary use of their houses, which is a more rational option as the Uygur merchant group is highly mobile under the market influence. Therefore, although centralized public rental housing is a community program led by the local government targeting management of the migrant population, “building a community around the market” still constitutes the deep logic that affects overall planning. At the same time, according to actual difficulties of ethnic minority groups, the local government actively intervenes and utilizes equal public services as a starting point. Such actions are highly significant in breaking down spatial barriers between different ethnic groups and greatly improving the working and living status of ethnic minority groups in the local area, creating the conditions for their exchanges, communication, and integration with indigenous residents. This community-building experience is in fact important aspect in the construction of an inter-embedded multi-ethnic community.

Therefore, although a highly-condensed Uygur community may seem to be an ethnic spatial aggregation, it is different in essence. Although the Uygurs live in the same community, their businesses are embedded in the overall jade market and they maintain close ties with all other stages of the local jade industry. Such embedded livelihood relationships form the basis for Uygur merchants to communicate and exchange with other ethnic groups in their local areas, and the community as a living space is the social extension of the market. The community mode of “living around the market" is an inter-woven community structure that differs from the aforementioned spatially-segregated ethnic community. It emphasizes all-around interactions by means of livelihood, lifestyles and spiritual cultures rather than maintenance of a relatively isolated community pattern.

## Conclusion

At present, the influx of ethnic minorities from China’s border area to other parts of China is expanding, the migration scope is constantly enlarging and ethnic minorities’ interaction with local society is increasing. The formation of urban multi-ethnic communities in various forms is bound to become a trend. However, the logic behind the formation of different forms of multi-ethnic communities may vary widely and therefore requires specific study.

From the experience of the Uygur community in Stone Town, the formation and development of this community is deeply embedded in the national jade market structure. It would be impossible to construct a migrant Uygur community without such a market structure. Conversely, a jade market with national influence could not be shaped in Stone Town without such an inter-embedded structure. It is precisely due to the joint involvement of many differentiated groups such as Uygur jade merchants that this embedded market structure can be maintained. Operating conditions and market demand are important factors in determining whether people will convene locally. The generally sluggish jade market in recent years has in fact forced some Uygur merchants to leave Stone Town to seek opportunities in other cities. Therefore, the degree of population aggregation is volatile in the context of market-induced mobility. Such multi-ethnic communities on the move are fundamentally different from ethnically segregated communities where residents live stably in tradition.

The core of this type of inter-embedded multi-ethnic community is not just the embedded living spaces, but the substantive embedding of the livelihood relationships behind them. The key to creating an inter-embedded multi-ethnic community is to explore these inter-embedded livelihood relationships and set them as the basic guarantee for the exchanges and interactions among different ethnic groups. In the case of Stone Town, the relatively independent living space is a result of local government planning and can essentially better adapt to embedded livelihood relationships rather than the other way around.

In addition, the limited interaction originating in the market among different ethnic groups will gradually develop into comprehensive interactive relationships with deepening mutual contact in more fields. The government’s active intervention and efforts to create conditions for this group to enjoy equal public services are critical manifestations of the construction of an inter-embedded multi-ethnic community. This is of great significance in promoting the exchange, communication, and integration of all ethnic groups. As ethnic minority groups possess particularities in terms of social and cultural life, if there is no governmental guidance or coordination in a highly heterogeneous society in the early stage, the migrant groups will encounter difficulties in finding reasonable solutions to various demands. They can only seek temporary help inside the group and cannot solve the problem at its core, which will inevitably aggravate alienation and estrangement between migrant groups and local society in the long run. Therefore, it is clear that by providing ethnic minority groups with more channels through which to deal with matters in life and improving their capabilities, encouraging the locals to actively participate in this course, and offering equal public services for this group, active intervention by the government can be an effective method of enhancing the exchanges and interactions among different ethnic groups.

Steady improvement of the common living environment and rich experience of getting along with each other is conducive to the sublimation of benign exchanges between individuals of different ethnic groups into extensive and in-depth exchange, communication, and integration among all ethnic groups. Interaction between ethnic groups is often presented through the individual relationship in concrete social life, and the relationship at the individual level in turn affects cognition and interaction between different groups. In the process of common pursuit of a better life, members of different nationalities will gradually transform estrangement and misunderstanding into mutual tolerance, understanding and inclusiveness. Benign individual relationships with rich connotations will be established with growing opportunities for mutual contact and accumulating experience of living together. Tacit understanding based on common values will be gradually cultivated and then be sublimated into extensive and in-depth exchange, communication, and integration among all ethnic groups.

Cross-regional population migration is a process of population redistribution based on the improvement of productive forces and the adjustment of production relations. It is an important manifestation of Chinese society at a period of important change, during which all social relations, including ethnic relations, will be naturally reshaped. The formation of an urban multi-ethnic community is an important form of ethnic minorities’ influx into the city, and is bound to be a critical link that will affect the evolution of ethnic relations in China in the future. The community form of “living around the market" is worthy of attention. However, more in-depth research should be conducted on how this form affects the reshaping of ethnic relations.

## Data Availability

Not applicable.

## Footnotes

1 Referring to Hu Jintao: “The 5^th^ Symposium on Tibetan Work where Hu Jintao put forward 7 points of “more emphasis”, Xinhua News Agency, January 22, 2010; “Xi Jinping presided over the 2^nd^ Central Symposium on Xinjiang Work”, Xinjiang, China, July 11, 2017; State Ethnic Affairs Commission: “The Spirit of the Central Conference on Ethnic Affairs Study Guidance Reader”, Ethnicity Press, 2015 edition, p. 107; Xi Jinping: “A decisive victory in building a moderately prosperous society in all aspects to seize the great victory of Socialism with Chinese Characteristics in the New Era - Report at the 19^th^ National Congress of the Communist Party of China”, People’s Daily, Oct. 28, 2017; Xi Jinping: “Promoting high-quality development of the Party’s ethnic work in the new era”, Xinhua News Agency, August 28, 2021.

2 The core meaning of the official statement includes: “The ethnic groups of China, including those in Xinjiang, live together alongside each other. They are economically interdependent and embrace each other's culture, and are a unified whole that has become impossible to separate. They are members of the same big family (of the Chinese nation)”. Source: "Historical Matters Concerning Xinjiang" by The State Council Information Office of the People's Republic of China, July 2019. https://www.fmprc.gov.cn/ce/cegv/eng/ztjs/aghj12w/t1914553.htm

3 The vast majority of ethnic minority migrants from Xinjiang engaged in jade trading in Nanyang are Uyghurs, with a small number of Hui. This study focuses on the Uyghur migrants.

4 Stone Town is under a pseudonym.

5 The demographic data on the Uyghur population in the Stone Town was compiled by the author during the 2016-2017 study based on oral interviews with relevant local government staff.

6 To protect the privacy of the interviewees, the real names of all interviewees and people in the cases are anonymized in this paper.

7 The service work includes joining in one “art performance”, convening one “symposium and exchange”, holding one “parents’ meeting”, having one “greeting visit”, carrying out one “sports competition”, organizing one “visit and exchange”, launching one “speech contest”, conducting one “family visit”, and launching one “publicity campaign”.
